# Neutrophil Gelatinase-Associated Lipocalin in Kidney Transplantation Is an Early Marker of Graft Dysfunction and Is Associated with One-Year Renal Function

**DOI:** 10.1155/2013/650123

**Published:** 2013-10-31

**Authors:** Isabel Fonseca, José Carlos Oliveira, Manuela Almeida, Madalena Cruz, Anabela Malho, La Salete Martins, Leonídio Dias, Sofia Pedroso, Josefina Santos, Luísa Lobato, António Castro Henriques, Denisa Mendonça

**Affiliations:** ^1^Department of Nephrology and Kidney Transplantation, Centro Hospitalar do Porto, Hospital de Santo António, 4099-001 Porto, Portugal; ^2^Unit for Multidisciplinary Investigation in Biomedicine (UMIB), 4099-003 Porto, Portugal; ^3^Department of Chemical Chemistry, Centro Hospitalar do Porto, Hospital de Santo António, 4099-001 Porto, Portugal; ^4^Institute of Public Health (ISPUP), University of Porto, 4050-600 Porto, Portugal; ^5^Department of Population Studies, Institute of Biomedical Sciences Abel Salazar (ICBAS), University of Porto, 4050-313 Porto, Portugal

## Abstract

Urinary neutrophil gelatinase-associated lipocalin (uNGAL) has been suggested as potential early marker of delayed graft function (DGF) following kidney transplantation (KTx). We conducted a prospective study in 40 consecutive KTx recipients to evaluate serial changes of uNGAL within the first week after KTx and assess its performance in predicting DGF (dialysis requirement during initial posttransplant week) and graft function throughout first year. Urine samples were collected on post-KTx days 0, 1, 2, 4, and 7. Linear mixed and multivariable regression models, receiver-operating characteristic (ROC), and areas under ROC curves were used. At all-time points, mean uNGAL levels were significantly higher in patients developing DGF (*n* = 18). Shortly after KTx (3–6 h), uNGAL values were higher in DGF recipients (on average +242 ng/mL, considering mean dialysis time of 4.1 years) and rose further in following days, contrasting with prompt function recipients. Day-1 uNGAL levels accurately predicted DGF (AUC-ROC = 0.93), with a performance higher than serum creatinine (AUC-ROC = 0.76), and similar to cystatin C (AUC-ROC = 0.95). Multivariable analyses revealed that uNGAL levels at days 4 and 7 were strongly associated with one-year serum creatinine. Urinary NGAL is an early marker of graft injury and is independently associated with dialysis requirement within one week after KTx and one-year graft function.

## 1. Introduction

Delayed graft function (DGF) is an important complication of kidney transplantation (KTx) that adversely affects allograft survival. Despite substantial improvements in the field of KTx, the incidence of DGF is rising with the growing practice of accepting expanded criteria donors to increase transplantation rates [1–6]. Delayed graft function predisposes kidney graft to acute and chronic rejection, contributes to progressive allograft dysfunction, and increases the risk of premature graft loss [7–11].

Reliable biomarkers enabling early discrimination of DGF in KTx are lacking, which impairs timely therapeutic interventions. Traditionally, acute graft dysfunction is diagnosed by measuring serum creatinine, but this parameter is an unreliable indicator of kidney function during an episode of acute injury [12]. One of the most promising biomarkers of acute kidney injury is neutrophil gelatinase-associated lipocalin (NGAL), which is released to blood from activated neutrophils during inflammatory processes. In steady situations, this lipocalin is found in urine only in trace. Massive NGAL quantities excreted in urine (uNGAL) usually indicate damage of proximal tubular cells [13–15]. Graft injury due to ischemia-reperfusion is an inevitable consequence of KTx procedure and can result in varying degrees of early graft dysfunction, which can be considered a form of posttransplantation acute kidney injury. For this reason, several studies investigated the utility of NGAL for the diagnostic and prognostic of acute graft dysfunction following KTx [16–27]. Recently, the prognostic value of uNGAL on graft function at one-year after transplantation was also examined and presented conflicting findings [22, 28].

In order to support the usefulness of uNGAL as a reliable marker of graft injury and to clarify the role of this promising biomarker in the prediction of kidney function beyond the first week after transplant, we conducted a prospective study to

- evaluate longitudinal changes of uNGAL levels over the first week after KTx and identify factors associated with these changes;
- assess the performance of uNGAL in predicting DGF (defined as the requirement for dialysis within the first 7 days after transplantation);
- appraise the long-term prognostic value of uNGAL measured within one week posttransplantation on kidney allograft function, evaluated by one-year serum creatinine.

## 2. Subjects and Methods

Consecutive patients with end-stage renal disease, undergoing living or deceased KTx at Centro Hospitalar do Porto, from December 2010 to May 2011 were prospectively enrolled. Recruitment excluded patients less than 18 years old and those who required a combined pancreas or liver KTx. After transplant, patients with primary graft failure related to surgical causes were excluded. This study was approved by Institutional Review Board of Centro Hospitalar do Porto. Each participant provided written informed consent before enrolment.

### 2.1. Sample Collection and Measurements

Urine samples for NGAL determination were collected 3 to 6 h after surgery (uNGAL0 or baseline); on the following morning, nearly 8 to 12 h after graft reperfusion (uNGAL1 or first day); and then at second (uNGAL2), fourth (uNGAL4), and seventh days (uNGAL7), for a total of five samples for each patient. The same laboratory technician, who was blinded to patient information, performed uNGAL measurements using a two-step chemiluminescent microparticle immunoassay on a standardized clinical platform (ARCHITECT, Abbott Diagnostics).

Serum creatinine levels were determined preoperatively, daily until hospital discharge, and at 1, 3, 6, and 12 months after transplantation to evaluate later graft function. Serum creatinine measurements were performed by Jaffé method (Roche Diagnostics). Cystatin C was measured with a particle enhanced immunonephelometric method (Siemens Diagnostics) at the same time points as uNGAL, except for baseline.

### 2.2. Definitions

*Delayed graft function* was defined, according to United Network for Organ Sharing, as the requirement for dialysis within the first seven days after KTx, due to an absence or irrelevant improvement in graft function. Complementarily, graft function was considered “*prompt*” (non-DGF) if no dialysis was required during the first week after transplantation.

*Acute Rejection* was defined as either biopsy-proven rejection or antirejection treatment without biopsy.

*Estimated Glomerular Filtration Rate (eGFR) *was calculated using the Rule's refitted MDRD formula [29], considered to have an improved diagnostic performance and better accuracy of the true GFR in KTx recipients [30].

*Creatinine Reduction Rate* (%) was calculated as the difference between serum creatinine at day 2 (or day 4) and day 1, divided by serum creatinine at day 1, multiplied by 100.

*Graft function at one year *was evaluated by the average of the two serum creatinine levels measured closer to one year after KTx (e.g., by the average values seen at 12 and 13 months). It was thought that this would reflect more accurately the usual graft function, since a single measure could be more easily inflated by acute situations, like a urinary infection for example. Two grafts were lost at seventh and eighth months and the last serum creatinine presented by these patients prior to dialysis restart was considered as being the one-year creatinine.

### 2.3. Statistics

Kolmogorov-Smirnov test was performed to assess deviation from normal distribution. Quantitative variables were summarized as mean and standard deviation (SD), or as median and 25th–75th quartiles (interquartile range) for variables with skewed distribution. Categorical variables were reported as percentages.

Statistical analysis was performed in five steps. Firstly, a cross-sectional bivariate analysis was done to compare groups and study the association between uNGAL and demographic/clinical variables. Continuous variables were compared using either parametric (*t*-test) or nonparametric (Mann-Whitney) tests. Associations between categorical variables were analyzed using the *χ* ^2^ test and Fisher's exact test as appropriate. Correlations between uNGAL and continuous variables were assessed using Pearson correlation and uNGAL levels were log-transformed (ln) before analysis. Spearman correlation was used to analyze uNGAL and serum creatinine reduction ratio on posttransplant days 2 and 4.

Secondly, we used a longitudinal analysis to study uNGAL kinetics and modelling it as a response variable on time. A linear mixed-effects model was used to study the association of DGF with serial changes of uNGAL (log-transformed), controlling for donor status (living/deceased), recipient's age, time on dialysis, and time measurement of uNGAL. The interaction between DGF and uNGAL time measurement was included in the model, as such a significant interaction would suggest that DGF affects the uNGAL levels trajectory.

Thirdly, receiver-operating characteristics analysis was performed to estimate the sensitivity and specificity of uNGAL (as well as serum creatinine and cystatin C) to predict DGF. The optimal cut-off points were determined by maximizing the sum of sensitivity and specificity.

Fourthly, multivariable logistic regression analysis was undertaken to evaluate whether uNGAL levels were independently associated with DGF. Pretransplant variables known to be associated with DGF and considered potential confounders were included in the models. To avoid collinearity each time point uNGAL was included separately in different models. The final models were fitted using a backward selection procedure.

Fifthly, multivariable linear regression was used to describe the independent association of uNGAL with renal function at 12 months evaluated by serum creatinine, adjusted for the variables that usually predict graft function, including donor status, rehospitalizations, and acute rejection episodes throughout the first year. Linear regression models used log-transformed uNGAL and serum creatinine levels. As in logistic models, uNGAL at each time point were included separately in models to avoid collinearity.

All statistical analyses were done with SPSS version 20.0 and a significance level of 0.05 was considered.

## 3. Results

During time recruitment, 42 patients were enrolled. Two recipients had renal artery thrombosis and were excluded in the first two posttransplantation days. Therefore our study sample included 40 recipients. Baseline data are shown in Table 1.

### 3.1. Urinary NGAL

The first urine sample (uNGAL0) was obtained from 30 patients. On the following days, urine samples were collected from 35 patients at the first, second, and seventh days, and from 36 patients at the fourth day. All of our subjects provided at least two urine samples. Only, one patient provided merely two urine samples and the remaining 39 subjects provided 3 or more urine samples (with 20 patients providing all five samples).

Daily median uNGAL levels did not differ significantly between male and female recipients, except for the seventh day where female uNGAL levels were significantly higher. Concerning donor status, uNGAL levels were higher in deceased donor recipients at all-time points, but only statistically significant at second day. Except for the seventh day, uNGAL levels were significantly and positively correlated with cold ischemia time (*r* = 0.45, *P* = 0.02; *r* = 0.36, *P* = 0.04; *r* = 0.56, *P* = 0.001; *r* = 0.46, *P* = 0.006, resp., at baseline, first, second, and fourth days). 

At most time points, uNGAL was positively and significantly correlated with recipient age (*r* = 0.39, *P* = 0.02; *r* = 0.39, *P* = 0.02; *r* = 0.44, *P* = 0.007; resp., at first, second, and seventh days) and pretransplant dialysis time (*r* = 0.48, *P* = 0.008; *r* = 0.37, *P* = 0.03; *r* = 0.43, *P* = 0.01; *r* = 0.33, *P* = 0.024; resp., at baseline, first, second, and seventh days). No significant correlation was found with HLA mismatches and with donor age and serum creatinine.

Urinary NGAL levels were significantly and positively correlated with serum creatinine at all-time points (data not shown). Furthermore, except for uNGAL0, all the remaining uNGAL levels were significantly and negatively correlated with changes in serum creatinine between the second and first days, and also between the fourth and the first days: lower uNGAL values were associated with higher reductions rates in serum creatinine (data not shown).

Median length of hospitalization after transplantation was 12 days (IQR: 7–22) and uNGAL levels were highly correlated with length of hospital stay at all-time points (*r* = 0.48, *P* = 0.002; *r* = 0.64, *P* < 0.001; *r* = 0.79, *P* < 0.001; *r* = 0.77, *P* < 0.001; *r* = 0.82, *P* < 0.001, resp., at baseline, first, second, fourth, and seventh days).

### 3.2. DGF and uNGAL Longitudinal Changes

Eighteen recipients (45%) had DGF, three of these were from living donors, and 22 (55%) had prompt graft function. Concerning traditional predictors of DGF and except for cold ischemia time, no significant differences were found between DGF/non-DGF in relation to baseline characteristics and induction therapy (Table 1). Mean age was significantly higher in patients with DGF (56 (11) versus 43 (16) years in non-DGF recipients, *P* = 0.006). As expected, patients with DGF had higher serum creatinine levels (Table 2) and lower creatinine reduction ratios on posttransplant days 2 and 4.

**Figure 1:**
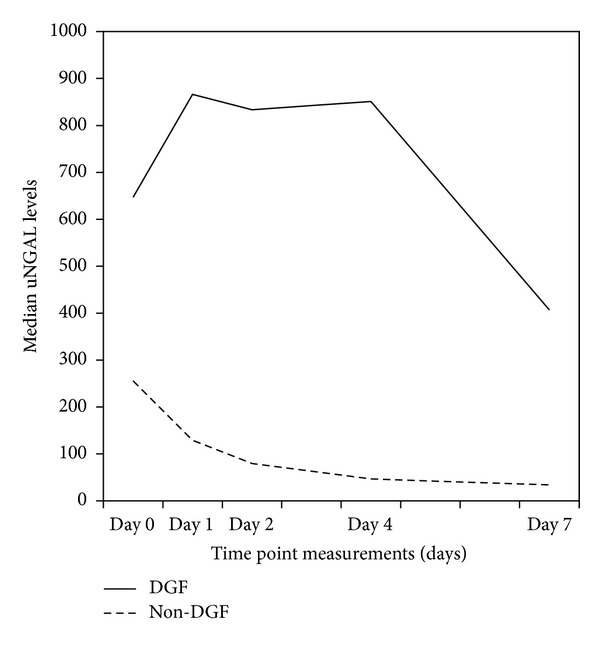
Evolution of uNGAL levels through first week after transplantation, according to graft function. Abbreviations: uNGAL: urinary neutrophil gelatinase associated lipocalin; DGF: delayed graft function.

Similar to serum creatinine, median uNGAL concentrations were consistently higher in DGF group compared with non-DGF group at all measured time points (Table 2 and Figure 1). In patients with prompt graft function, the longitudinal changes of uNGAL were characterized by an initial phase with a rapid decline and then a phase with a slower decrease continuing throughout the posttransplant week. This pattern of changes was different in DGF recipients: uNGAL levels increased from baseline to the following morning after transplantation and remained elevated throughout most of the follow-up period.

**Figure 2:**
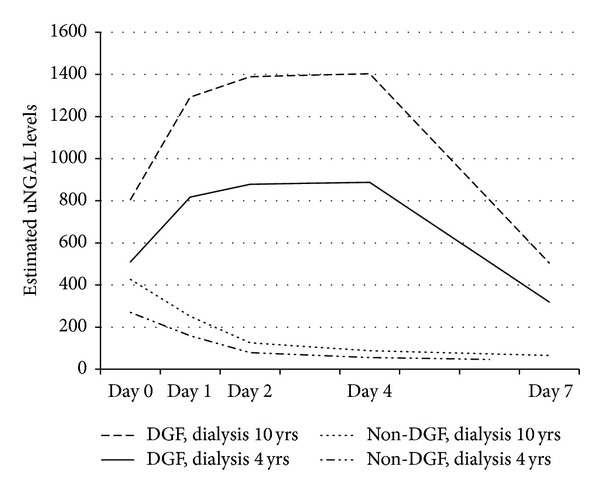
Predicted uNGAL values over time of four hypothetical subjects, estimated from multiple linear mixed model presented in Table 3. Abbreviations: uNGAL: urinary neutrophil gelatinase associated lipocalin; DGF: delayed graft function; non-DGF: prompt graft function.

A linear mixed-effects model was used to study the association of DGF with longitudinal changes of uNGAL, controlling for variables found to be associated with uNGAL by bivariate analysis. Pretransplant time on dialysis, time measurement of uNGAL, and DGF were independently associated with uNGAL levels. Adjusting for the remaining variables, donor status and recipient age lost their statistical significance and were removed from the final model (Table 3). Delayed graft function was significantly associated with uNGAL levels, with prompt function recipients having on average lower levels of uNGAL at all-time points. According to our estimation, for a patient with dialysis time of approximately 4.1 years, the initial values of uNGAL (3–6 h after transplantation) are about 242 ng/mL higher in patients who went on to develop DGF, and these values will rise even more in the following days. A significant interaction between time of measurement and DGF confirmed that longitudinal changes of uNGAL levels depend on whether the recipient had DGF or not. To clarify the meaning of this interaction, Figure 2 shows the predicted uNGAL trajectories over time for four hypothetical subjects: two recipients who developed DGF (one with 4 years of dialysis and one with 10 years), and two other patients with prompt graft function (similar time on dialysis, 4 and 10 years). Hypothetically, the remaining variables were equal in all four patients. The predicted uNGAL values were estimated using the coefficients estimates of Table 3 (e.g., the predicted uNGAL values at the first day for a recipient with 4 years of dialysis with prompt function = exp [(5.46 − 2.14) + 0.94 + 0.4 + (0.076 ∗ 4 years of dialysis)] = 158 ng/mL).

### 3.3. Prediction of DGF by uNGAL Levels (ROC analysis)

**Figure 3:**
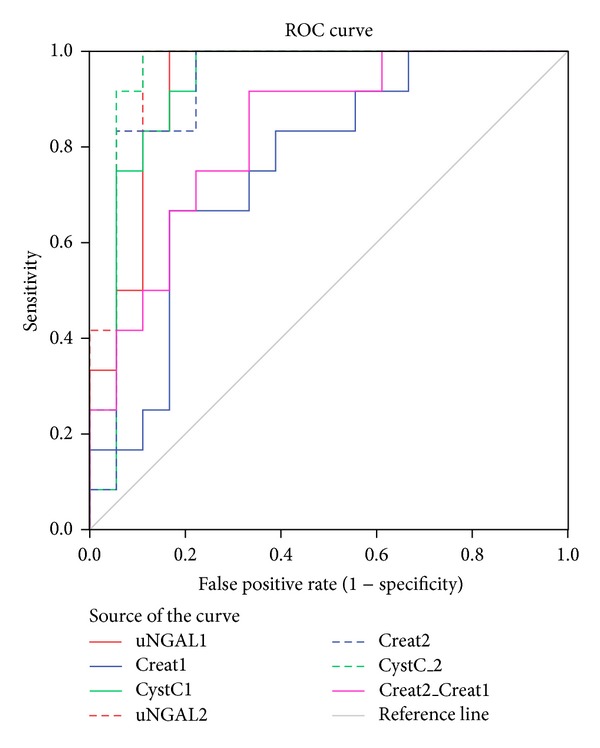
Receiver-operating characteristic curves for uNGAL, serum creatinine and changes in serum creatinine, and serum cystatin C measured at posttransplant days 1 and 2 for predicting delayed graft function. Abbreviations: uNGAL: urinary neutrophil gelatinase associated lipocalin; Creat: serum creatinine; Cyst: serum Cystatin C; Creat2-Creat1: creatinine reduction rate between the first and the second day.

Receiver-operating characteristic (ROC) curves showed uNGAL on the first postoperative days were accurate in predicting DGF (Tables 4 and 5).  Table 4 displays the derived sensitivities, specificities, and predictive values for uNGAL at the cutoff concentrations that provided the maximum sum of sensitivity and specificity. Regarding the areas under the ROC curves (AUC), the ability of uNGAL to predict DGF was moderately accurate at baseline and first day, and highly accurate at second, fourth, and seventh days (Table 5, Figure 3). In the first two posttransplant days the diagnostic performance of uNGAL was better than of serum creatinine, and quite similar to that of cystatin C. The reduction in serum creatinine between first and second days resulted in AUC = 0.78 [0.64–0.92] and was worse than uNGAL for DGF prediction.

### 3.4. Independent Association of uNGAL Levels and DGF (Multivariable Analyses)

Multivariable logistic regression analyses revealed that uNGAL levels remained independently associated with DGF at most time points, after adjusting for clinically relevant risk factors for DGF (Table 6). Furthermore, recipient age was the other significant independent predictor of DGF in almost all models. To be more clinically relevant, estimates of DGF risk were converted to every 50 ng/mL of increase in uNGAL or per each 5 years of increase in age, instead of estimates per each unit of increase.

### 3.5. Within One-Year after Kidney Transplantation

During the first year, 10 KTx recipients were rehospitalized accounting for a total of 19 hospital admissions. There was one rehospitalization in six patients, two in two patients, three in one patient, and six rehospitalizations in one patient with a psychological disorder and suicidal ideation. The causes of rehospitalization were infection in five admissions (mostly, urinary tract infection), renal dysfunction in six, and nonrenal causes in the remaining eight admissions (suicidal ideation, acute pulmonary edema, and neutropenia).

Excluding the recipient with several rehospitalizations due to psychological decompensation, the length of hospital stay of the remaining recipients admissions was 7 [3] days, and no significant differences were found between recipients from living or deceased donors.

The acute rejection episodes were collected throughout the first posttransplant year. Ten recipients (25%) had an acute rejection episode during inpatient hospitalization for transplant surgery, and only one patient was rehospitalized one month after KTx with an acute rejection episode confirmed by biopsy.

At one year after transplantation, all patients were alive but two grafts were lost. At this time, the median plasma creatinine was significantly higher in DGF group compared to non-DGF: 1.6 mg/dL [IQR: 1.2–2.5] versus 1.3 mg/dL [IQR: 1.0–1.5], *P* = 0.049. 

### 3.6. Prognostic Value of First-Week uNGAL Levels in One-Year Graft Function

The correlation between uNGAL collected in the first week after KTx and serum creatinine at one year was explored. Except for uNGAL collected within the first 24 h after transplantation, uNGAL levels were positively correlated with serum creatinine evaluated at time of discharge, and also at 1, 3, 6, and 12 months. Likewise, uNGAL levels at days 2, 4, and 7 were inversely correlated with eGFR at 6 and 12 months (data not shown).

The prognostic value of early uNGAL values on long-term allograft function (one year after KTx) was tested by multivariable analysis. In multivariable linear regression models for serum creatinine at 12 months, uNGAL measured on the fourth and seventh days were independently associated with one-year graft function, adjusting for established variables that usually affect graft function, including acute rejection episodes and rehospitalizations that occurred during the first posttransplant year (Table 7).

## 4. Discussion

The major finding of this study is that uNGAL is a promising biomarker for allograft dysfunction that can be easily and noninvasively assayed in the early posttransplant period. We prospectively evaluated uNGAL in a cohort of 40 kidney allograft recipients during the first posttransplant week. At all measured time points, uNGAL levels were consistently higher in patients who developed DGF, including the earliest levels obtained from the first urine sample collected approximately 3 to 6 h after transplant surgery. At this time, clinical diagnosis of DGF is yet not possible, but a simple and noninvasive test can already recognize kidney dysfunction and stratify patients according to likelihood of requiring posttransplant dialysis.

It would be ideal to diagnose graft dysfunction with an early and highly sensitive biologic marker of renal tubular injury. One of the most promising markers is NGAL, and our findings provide further information for the use of uNGAL as a diagnostic and prognostic tool for DGF. According to our estimation, uNGAL values shortly after transplant surgery will be much higher in patients who went on to develop DGF and will rise further in the following days. In contrast, patients with prompt function will have lower levels, which decrease consistently along the week. The kinetics of changes in these recipients compared to those who presented DGF is quite different. It seems that, not only the baseline levels, but also the pattern of uNGAL longitudinal changes can reflect graft dysfunction.

The association between higher NGAL levels and DGF after KTx has been previously published [16, 20, 22, 24, 25]. But the findings are not consistent regarding the kinetics of uNGAL according to DGF. Hollmen et al. [22] found initial levels of uNGAL higher in DGF patients, but on the following day a decrease was observed, as it happened with recipients with prompt function. As mentioned before, our study did not confirm this declining in DGF patients. Recipients who went on to develop DGF had initial higher levels of uNGAL that rise further on the following posttransplant days, differing from patients with prompt graft function. Our findings are in agreement with results reported by Hall et al. [24]. It seems that, above and beyond the markedly higher levels of uNGAL in patients with graft dysfunction, the contrasting pattern of uNGAL longitudinal changes can distinguish recipients who will need dialysis in the first week posttransplantation.

To the best of our knowledge, this is the first report that used linear mixed analysis in describing longitudinal changes of uNGAL in the first week following KTx. Multiple observations of a variable on a particular patient are likely to be positively correlated, so they should not be treated as independent measurements. Although models that take this design into consideration are more complicated, they are also more specific and powerful since they permit the study of changes over time. Linear mixed analysis not only permits to model individual changes over time, but also is able to distinguish within-subject from between-subject sources of variation [31].

In accordance with previously published data [16, 20–22, 24], we confirmed the good performance of NGAL in predicting graft dysfunction in the early posttransplant period. Using ROC analysis, our study also corroborates uNGAL as a good diagnostic marker on identifying patients with graft dysfunction and who subsequently required dialysis. The AUC-ROC for uNGAL was moderately accurate for DGF prediction within the first day after transplant, and it was excellent at day 2 and day 4. We also determined the paired sensitivity and specificity for the cutoff value of uNGAL, calculated to be closest to the left upper corner of the ROC space to predict DGF. At 8 to 12 h after surgery, a cutoff of 286 ng/mL had 100% sensitivity and 76% specificity for the identification of DGF. Within the second day, uNGAL levels higher than 277 ng/mL predicted DGF with a sensitivity of 93% and specificity of 90%. Other studies showed also impressive results. Parikh et al. [16] in a study that included 53 patients undergoing KTx, measured NGAL in urine samples collected within the first 24 h following transplantation and reported an AUC-ROC of 0.9, similar to ours obtained 8 to 12 h after surgery. Another study [24] conducted in 91 recipients evaluated uNGAL within 6 h after transplantation and predicted subsequent DGF with an AUC-ROC = 0.81. Most recently, Hollmen et al. [22] undertook a large cohort study that included 176 KTx recipients. Urine was collected before transplant, at then at days 1, 3, 7, and 14, and uNGAL was measured at each time point. The authors found and AUC-ROC = 0.74 at day 1.

We report a superior performance of uNGAL level for predicting DGF over serum creatinine measured at the same time. Urinary NGAL measured at the first day predicted DGF with an AUC-ROC of 0.93, which is markedly better than an AUC-ROC = 0.76 shown by serum creatinine measured in the same day, and also than an AUC-ROC = 0.83 obtained from creatinine reduction ratio from first to second day, but quite similar to cystatin C (0.95), a marker considered more accurately to detect changes in renal function [32–35]. Furthermore, our analyses also revealed that uNGAL levels predicted DGF, even after adjusting for pretransplant variables known to be traditionally associated with DGF.

Besides DGF, the other factors that significantly influenced uNGAL levels were previous time on dialysis, recipient's age at time of transplantation and cold ischemia time. These three variables were positively correlated with uNGAL values. Mishra and coworkers [17] have shown that the immunohistochemical staining intensity for NGAL was strongly correlated with cold ischemia time and NGAL expression was significantly increased in deceased donor biopsies. We found that uNGAL levels were higher in graft recipients from deceased donors, but only significantly higher at the second day. It is known that prolonged cold preservation of kidneys can lead to severe injury, which is critical in the success of deceased-donor kidney transplantation [36]. However, there is a progressive effort of our transplant team to avoid prolonged cold preservation. Maybe this attempt attenuated the effect of cold ischemic injury in kidneys from deceased donors, which become comparable to living donors concerning uNGAL values. An interesting finding of our study was that uNGAL levels at all-time points were correlated with length of hospital stay. It is well known that the occurrence of DGF prolongs the recipient's hospital stay. And it is worthy of note to realize that patients with early higher levels of uNGAL will expect longer time of hospitalization, probably due to graft dysfunction.

As other studies [16–19, 24, 26, 37], we confirmed that uNGAL levels were inversely correlated with eGFR and positively correlated with serum creatinine at each measured time point. We also showed that not only in the first week, but longer after that, uNGAL levels measured in the first seven days after KTx were still predictive of graft function throughout the first year after transplantation. Even after adjusting for donor status, acute rejection episodes, hospitalizations occurred in the first year, and other known variables that usually affect graft function, uNGAL evaluated at days four and seven were predictive of one-year serum creatinine, which can be considered a surrogate marker of long-term graft survival [38, 39]. In contrast, Hollmen and coworkers study [22] did not find any correlation between uNGAL and renal function at one year. In their study, uNGAL collected in the first two weeks after transplantation was only correlated with renal function up to 3 months. Our results do not corroborate this lack of correlation and are in agreement with a recent study that also associated perioperative uNGAL levels to one-year allograft function [28].

Our study has several strengths. First, it is a prospective-cohort design study. Second, we measured uNGAL at several time points within the first posttransplant week, and not at one single point. Longitudinal studies have the advantage of providing detailed information about how a marker changes over time; however the studies present some statistical complexities, since the customary assumption that all observations are independent usually does not hold. And this was the third strength of our study: the use of longitudinal methods to handle the serial changes of uNGAL. A fourth strength was the technical determination that we have chosen to measure NGAL. We used a commercially available kit for uNGAL determination (Abbott Architect NGAL), which is simple to implement in routine practice and it is considered one of the best methods for detecting acute kidney injury [40].

Similarly to other authors [16, 22], we have chosen to measure NGAL in urine, instead of blood, since uNGAL represents tubule damage in the kidney rather than filtration from blood [14, 41]. An increased level of NGAL in urine usually indicates injury of proximal tubular cells and seems to be more specific compared to serum NGAL, which can be produced by other organs and released into the circulation following a transplant surgery [42]. Other advantages of urinary diagnostics include the noninvasive nature of sample collection and the reduced number of interfering protein [43]. However, despite the undoubtedly value of urinary markers of kidney injury, their use in transplant recipients can be also a drawback because of possible transient graft anuria, which may preclude the availability of urine and consequently the lack of sample to measure NGAL. Due to the shortcoming of urine biomarkers in anuric KTx recipients, some studies have evaluated the performance of serum/plasma NGAL in predicting graft function recovery after KTx [21, 27]. As we did not measure serum/plasma NGAL values, we could not compare their effectiveness in predicting DGF in our sample. In our study, 4 or 5 recipients were anuric in each measurement, resulting in 12% of our patients not having urine sample to determine uNGAL in that particular time point. The measurement of serum/plasma NGAL could have been a valuable alternative in these recipients, since it could also be obtained noninvasively in patients who required dialysis during the transient period of anuria.

Similar to other areas in medicine, in kidney transplantation early diagnosis and timely intervention will improve outcomes. Ischemic injury of the renal allograft is a critical early insult that increases the risk of acute tubular necrosis and long-term graft loss. If DGF could be detected in the early hours after surgical procedure, maybe a tailored and more individualized intervention could be achieved. Perioperative fluid management must ensure the restoration and maintenance of the intravascular volume, in order to obtain an appropriate graft function. Aggressive hydration has been recognized to be effective in avoiding DGF, but fluid overload may also precipice the need of dialysis with the risk of hipovolemia and consequent renal ischemia. Early identification of DGF patients could allow to be more judicious and to modify postoperative fluid management in favor of maintaining just adequate filling pressures to maintain adequate intravascular volume and prevent fluid overload [44]. Regarding immunosuppression, the induction protocol chosen for this group of patients should have the associated effect of decreasing DGF rates, by suppressing leukocyte-rich vascular congestion and endothelial injury, and the introduction of calcineurin inhibitors could be avoided or delayed due to their vasoconstrictive properties. Cytomegalovirus (CMV) infection has also direct and indirect effects on transplant graft function, and some previous evidence has been published relating the association between the use of ganciclovir and the lower occurrence of DGF [45]. Nowadays, the prophylaxis with valganciclovir should be other aspect taken into account in recipients that we know they will develop DGF, since this prophylaxis may do more than just delay the occurrence of CMV disease.

Several studies were done in renal transplantation to identify early biomarkers for the diagnosis of DGF. However, there is still no routine application of any of these markers in clinical transplantation. The present study clearly support that uNGAL represents an early marker of graft injury and is strongly associated with dialysis-based diagnosis of DGF and one-year graft function. Other studies are necessary to clarify the genesis and sources of plasma and urinary NGAL and validate the accuracy of uNGAL as a diagnostic marker of renal graft injury and predictor for DGF in assorted centres, across different practices and sets of variables.

## Figures and Tables

**Table 1:** Summary of baseline and clinical characteristics in kidney transplant donors and recipients (total sample and categorized by delayed or prompt graft function).

	Total (*n* = 40)	DGF (*n* = 18)	Non-DGF (*n* = 22)	*P* value
Donor				
Age (yr)	51.2 ± 11.4	51.1 ± 13.4	51.2 ± 9.9	0.172
Male gender	26 (65)	14 (78)	12 (54.5)	0.125
Living donor	11 (27.5)	3 (16.7)	8 (36.4)	0.165
Expanded criteria donors	3 (7.5)	1 (5.6)	2 (9.1)	0.541
Serum creatinine (mg/dL)	0.81 ± 0.18	0.85 ± 0.21	0.78 ± 0.16	0.318
Donor-recipient				
HLA mismatches	3.39 ± 1.24	3.38 ± 1.07	3.41 ± 1.46	0.941
Cold ischemia time (h)	12.1 ± 7.9	15.2 ± 7.8	9.6 ± 7.3	0.035*
Living donor	2.8 ± 0.5	2.5 ± 0.5	3.0 ± 0.5	0.204
Deceased donor	16.2 ± 5.9	18.1 ± 5.1	14.1 ± 6.2	0.088
Recipient				
Age (yr)	49.2 ± 15.2	56.3 ± 10.9	43.3 ± 15.9	0.006*
Male gender	26 (65)	11 (61)	15 (68)	0.641
Caucasian	40 (100)	18 (100)	22 (100)	—
BMI (Kg/m^2^)	24.8 ± 4.9	26.2 ± 4.4	23.6 ± 5.0	0.091
Previous transplant	2 (5)	0 (0)	2 (9.1)	
Time on dialysis (yr)	4.4 ± 4.7	5.6 ± 6.2	3.4 ± 2.3	0.135
Pretransplant therapy				
Dialysis	38 (95)	18 (100)	20 (90.9)	0.296
Preemptive transplantation	2 (5)	0 (0)	2 (9.1)	
Cause of kidney disease				
IgA nephropathy	7 (17.5)	2 (11.1)	5 (22.7)	—
Glomerulonephritis	6 (15.0)	4 (22.2)	2 (9.1)	—
Diabetic nephropathy	5 (12.5)	3 (16.7)	2 (9.1)	—
Autosomal dominant polycystic kidney disease	3 (7.5)	3 (16.7)	0 (0)	—
Unknown	4 (10.0)	1 (5.6)	3 (13.6)	—
Others	15 (37.5)	5 (27.8)	10 (45.5)	—
Peak PRA (%)	5.5 ± 15.1	5.0 ± 15.0	5.9 ± 15.5	0.853
0	29 (72.5)	14 (77.8)	15 (68.2)	—
1–25	8 (20.0)	3 (16.7)	5 (22.7)	—
26–75	3 (7.5)	1 (5.6)	2 (9.0)	—
Current PRA (%)	2.3 ± 8.6	3.1 ± 11.7	1.6 ± 4.9	0.585
0	34 (85)	15 (83.3)	19 (86.4)	—
1–25	5 (12.5)	2 (11.1)	3 (13.6)	—
26–50	1 (2.5)	1 (5.6)	0 (0)	—
Induction regimen				
Antithymocyte globulin (ATG-F)	4 (10)	1 (5.6)	3 (13.6)	0.613
Basiliximab/daclizumab	30 (75)	14 (77.8)	16 (72.7)	0.789
Immunosuppression at time of discharge				
Steroids	38 (95.0)	18 (100)	20 (90.9)	0.296
Tacrolimus	38 (95.0)	17 (94.4)	21 (95.5)	0.886
Cyclosporine A	2 (0.05)	1 (5.6)	1 (5.6)	0.884

Values are expressed as mean ± standard deviation or absolute numbers and percentages. Comparisons between continuous variables were done using parametric (*t*-test) or nonparametric (Mann-Whitney) tests; associations between categorical variables were analyzed using the *χ* ^2^ test and Fisher's exact test as appropriate; *P < 0.05.

Abbreviations: HLA: human leukocyte antigen; BMI: body mass index; PRA: panel reactive antibody.

**Table 2:** Serial levels of serum creatinine and uNGAL through the first posttransplant week, according to graft function (delayed or prompt).

Serum Creatinine (mg/dL) Median, (IQR)	Prior transplantation	1st day* (*n* = 40, 18 DGF)	2nd day (*n* = 40, 18 DGF)	4th day (*n* = 40, 18 DGF)	7th day (*n* = 40, 18 DGF)
DGF (*n* = 18)	7.5 (6.0–11.7)	8.2 (6.5–9.3)	7.5 (5.9–8.5)	6.9 (6.1–8.0)	6.4 (5.3–8.9)
Non-DGF (*n* = 22)	7.8 (5.1–9.4)	6.3 (4.6–7.9)	4.3 (2.8–6.1)	2.5 (1.6–3.2)	1.9 (1.4–2.4)

Urine NGAL (ng/mL) Median, (IQR)	3 to 6 h after surgery (*n* = 30, 13 DGF)	1st day* (*n* = 35, 14 DGF)	2nd day (*n* = 35, 15 DGF)	4th day (*n* = 36, 15 DGF)	7th day (*n* = 35, 16 DGF)

DGF (*n* = 18)	647 (328–1648)	866 (500–1256)	834 (510–2632)	851 (549–1643)	407 (106–1249)
Non-DGF (*n* = 22)	256 (105–446)	129 (64–306)	80 (29–138)	47 (36–91)	34 (26–57)

*1st day = 8 to 12 h after surgery; values are medians and interquartile range (25th to 75th percentile).

Abbreviations: uNGAL: urinary neutrophil gelatinase associated lipocalin; IQR: interquartile range; DGF: delayed graft function; non-DGF: prompt function.

**Table 3:** Results of the final linear mixed model for dependent variable ln(uNGAL) (*n* = 171 observations derived from 40 patients).

	Coefficient estimate	*P *value	95% CI	
Intercept	5.46	<0.001	4.94	5.98
Graft function				
DGF = 0 (prompt graft function)	−2.04	<0.001	−2.64	−1.44
DGF = 1 (with DGF-reference)	0	—	—	—
Time				
Time (3 to 6 h after surgery)	0.47	0.088	−0.07	1.00
Time (1st day)	0.94	0.001	0.41	1.47
Time (2nd day)	1.01	<0.001	0.49	1.53
Time (4th day)	1.02	<0.001	0.50	1.54
Time (7th day-reference)	0	—	—	—
Time ∗ DGF				
Time (3 to 6 h after surgery) ∗ DGF = 0	1.40	<0.001	0.68	2.13
Time (1st day) ∗ DGF = 0	0.40	0.257	−0.29	1.10
Time (2nd day) ∗ DGF = 0	−0.37	0.295	−1.06	0.32
Time (4th day) ∗ DGF = 0	−0.73	0.039	−1.42	−0.03
Time (7th day) ∗ DGF = 0 (reference)	0	—	—	—
Time on dialysis	0.076	0.003	0.03	0.12

Abbreviations: uNGAL: urinary neutrophil gelatinase associated lipocalin; ln: natural logarithm; DGF: delayed graft function (DGF = 0, no delayed graft function).

**Table 4:** Sensitivity, specificity, and predictive values for DGF using specific uNGAL cut-off values.

Time after transplant	uNGAL cutoff (ng/mL)	Sensitivity (%)	Specificity (%)	PPV	NPV
Shortly after surgery (3 to 6 h)	479	77	88	87	79
1st day (8 to 12 h after surgery)	286	100	76	81	100
2nd day	277	93	90	90	93
4th day	232	93	95	95	93
7th day	63	94	84	86	93

DGF: delayed graft function; uNGAL: urinary neutrophil gelatinase-associated lipocalin; PPV: positive predictive value; NPV: negative predictive value.

**Table 5:** Area under the receiver-operating characteristic curve at each time point for uNGAL, serum creatinine, and serum cystatin C for predicting DGF.

	Time after transplant	AUC (95% CI)	*P* value
Urine NGAL (ng/mL)	Shortly after surgery (3 to 6 h)	0.77 (0.58–0.97)	0.010
	1st day (8 to 12 h after surgery)	0.88 (0.77–1.0)	<0.001
	2nd day	0.96 (0.90–1.0)	<0.001
	4th day	0.99 (0.97–1.0)	<0.001
	7th day	0.93 (0.86–1.0)	<0.001

Serum creatinine (mg/dL)	Prior transplantation	0.56 (0.38–0.74)	0.514
	1st day (8 to 12 h after surgery)	0.77 (0.61–0.93)	0.007
	2nd day	0.90 (0.79–1.0)	<0.001
	4th day	0.95 (0.87–1.0)	<0.001
	7th day	0.93 (0.81–1.0)	<0.001

Serum cystatin C (mg/L)	1st day (6 to 12 h after surgery)	0.90 (0.79–1.0)	<0.001
	2nd day	0.96 (0.88–1.0)	<0.001
	4th day	0.95 (0.89–1.0)	<0.001
	7th day	0.93 (0.83–1.0)	<0.001

DGF: delayed graft function; uNGAL: urinary neutrophil gelatinase-associated lipocalin.

**Table 6:** Association of uNGAL with delayed graft function by multivariable analysis (logistic regression).

	Delayed graft function		
	OR adjusted*	*P* value	95% CI
Model 1 (uNGAL at 3 to 8 h after surgery)			
uNGAL0 (per 50 ng/mL of increase)	1.15	0.044	1.01–1.31
Recipient age (per 5 years of increase)	1.49	0.054	0.99–2.24
Model 2 with (uNGAL at day 1)			
uNGAL1 (per each 50 ng/mL of increase)	1.22	0.012	1.05–1.42
Recipient age (per 5 years of increase)	1.99	0.022	1.11–3.57
Model 3 (uNGAL at day 2)			
uNGAL2 (per each 50 ng/mL of increase)	1.35	0.004	1.10–1.66
Model 4 (uNGAL at day 4)			
uNGAL4 (1 ng/mL increase)	3.01	0.035	1.08–8.40
Model 5 (uNGAL at day 7)			
uNGAL7 (per each 50 ng/mL of increase)	1.43	0.050	1.01–2.04
Recipient age (per 5 years of increase)	1.73	0.038	1.03–2.90

Note: results given by logistic regression (backward Wald test).

Abbreviations: OR: odds ratio; 95% CI (95% confidence interval); uNGAL: urinary neutrophil gelatinase-associated lipocalin.

*Adjusted for pretransplant time on dialysis, recipient gender and age, and donor age.

**Table 7:** Significant factors associated with serum creatinine at one year after kidney transplantation.

	Regression coefficient adjusted*	*P*-value	95% CI
Model with uNGAL at day 4			
Donor gender (male versus female)	0.042	0.004	0.015–0.069
Donor age (years)	0.011	0.008	0.003–0.020
uNGAL4 (ln, ng/mL)	0.067	0.045	0.002–0.132
Model with uNGAL at day 7			
Time on dialysis (ln, ng/mL)	0.042	0.004	0.015–0.069
Donor age (years)	0.018	0.002	0.008–0.029
uNGAL7 (ln, ng/mL)	0.138	0.007	0.041–0.235

Note: results given by multiple linear regression; serum creatinine (ln) at 12 months as the dependent variable. Only the significant variables associated with serum creatinine are displayed. Abbreviations: uNGAL: urinary neutrophil gelatinase-associated lipocalin.

*Adjusted for donor status, donor age, recipient age and gender, pretransplant time on dialysis, rehospitalizations, and acute rejection episodes throughout the first year.
